# Neonatal Neurobehavior and Diffusion MRI Changes in Brain Reorganization Due to Intrauterine Growth Restriction in a Rabbit Model

**DOI:** 10.1371/journal.pone.0031497

**Published:** 2012-02-08

**Authors:** Elisenda Eixarch, Dafnis Batalle, Miriam Illa, Emma Muñoz-Moreno, Ariadna Arbat-Plana, Ivan Amat-Roldan, Francesc Figueras, Eduard Gratacos

**Affiliations:** 1 Department of Maternal-Fetal Medicine, Institut Clinic de Ginecologia, Obstetricia i Neonatologia (ICGON), Hospital Clinic, Barcelona, Spain; 2 Institut d'Investigacions Biomèdiques August Pi i Sunyer (IDIBAPS), University of Barcelona, Barcelona, Spain; 3 Centro de Investigación Biomédica en Red de Enfermedades Raras (CIBERER), Barcelona, Spain; Hôpital Robert Debré, France

## Abstract

**Background:**

Intrauterine growth restriction (IUGR) affects 5–10% of all newborns and is associated with a high risk of abnormal neurodevelopment. The timing and patterns of brain reorganization underlying IUGR are poorly documented. We developed a rabbit model of IUGR allowing neonatal neurobehavioral assessment and high resolution brain diffusion magnetic resonance imaging (MRI). The aim of the study was to describe the pattern and functional correlates of fetal brain reorganization induced by IUGR.

**Methodology/Principal Findings:**

IUGR was induced in 10 New Zealand fetal rabbits by ligation of 40–50% of uteroplacental vessels in one horn at 25 days of gestation. Ten contralateral horn fetuses were used as controls. Cesarean section was performed at 30 days (term 31 days). At postnatal day +1, neonates were assessed by validated neurobehavioral tests including evaluation of tone, spontaneous locomotion, reflex motor activity, motor responses to olfactory stimuli, and coordination of suck and swallow. Subsequently, brains were collected and fixed and MRI was performed using a high resolution acquisition scheme. Global and regional (manual delineation and voxel based analysis) diffusion tensor imaging parameters were analyzed. IUGR was associated with significantly poorer neurobehavioral performance in most domains. Voxel based analysis revealed fractional anisotropy (FA) differences in multiple brain regions of gray and white matter, including frontal, insular, occipital and temporal cortex, hippocampus, putamen, thalamus, claustrum, medial septal nucleus, anterior commissure, internal capsule, fimbria of hippocampus, medial lemniscus and olfactory tract. Regional FA changes were correlated with poorer outcome in neurobehavioral tests.

**Conclusions:**

IUGR is associated with a complex pattern of brain reorganization already at birth, which may open opportunities for early intervention. Diffusion MRI can offer suitable imaging biomarkers to characterize and monitor brain reorganization due to fetal diseases.

## Introduction

Intrauterine growth restriction (IUGR) due to placental insufficiency affects 5–10% of all pregnancies and induces cognitive disorders in a substantial proportion of children [1]. Reduction of placental blood flow results in chronic exposure to hypoxemia and undernutrition [2] and this has consequences on the developing brain [3]. The association between IUGR and short- [4], [5] and long-term [4], [6]–[12] neurodevelopmental and cognitive dysfunctions has been extensively described. Additionally, magnetic resonance imaging (MRI) studies have consistently demonstrated brain structural changes on IUGR [13]–[17]. Decreased volume in gray matter (GM) [13] and hippocampus [14], and major delays in cortical development [15] have been reported in neonates, as well as reduced GM volumes [16] and decreased fractal dimension of both GM and white matter (WM) [17] in infants.

The development of imaging biomarkers for early diagnosis and monitoring of brain changes associated with IUGR is among the challenges to improve management and outcomes of these children. There is a need to improve MRI characterization of the anatomical patterns of brain reorganization associated with IUGR and to develop specific imaging biomarkers. In spite of previous studies the timing and pattern of brain abnormalities associated with IUGR is still ill-defined. The acquisition of high resolution MRI images is limited in fetuses and neonates due to size and motion artefact issues [18], [19]. In addition, there is some variability among MRI postnatal studies, which may be influenced by variations in the case definition used and the postnatal morbidity associated with IUGR [20]. Notwithstanding their obvious shortcomings, animal models may overcome some limitations of human studies. Aside from the reproducibility of experimental conditions, such settings permit performing MRI on isolated whole brain preparations, which allows increasing substantially the duration of acquisition time and hence, the use of high resolution acquisition approaches [21].

Contrary to acute perinatal events, IUGR is a chronic condition that induces brain reorganization and abnormal maturation rather than gross tissue destruction [22]. Consequently, it requires the use of MRI modalities allowing to identify subtle changes in brain structure. Among these, diffusion MRI offers a promising approach to assess abnormalities in brain maturation and develop biomarkers for clinical use [23]. Diffusion MRI measures the diffusion of water molecules in tissues and obtains information about brain microstructure and the disposition of fiber tracts [24]. Diffusion MRI has been consistently shown to be highly sensitive to changes after acute hypoxia in adults [25], [26] and developing brain [23], [27]. Aside from reflecting acute injury, diffusion MRI parameters seem to correlate well with brain maturation and organization in fetal and early postnatal life [23], [28]. In addition, preliminary clinical results suggest that diffusion MRI could also be suitable to detect maturational changes occurring in chronic fetal conditions, including fetal cardiac defects [29] and IUGR [30].

In this study we developed a rabbit model allowing to perform neurobehavioral tests and high resolution diffusion MRI. The fetal rabbit was selected for several reasons. Firstly, selective ligature of uteroplacental vessels in this model has been demonstrated to reproduce growth impairment and hemodynamic adaptation as occurring in human IUGR [31]–[33]. Secondly, the rabbit presents a human-like timing of perinatal brain WM maturation [34]. Finally, validated tests for the objective evaluation of neonatal neurobehavior are available [35]. In addition, we developed a protocol to perform diffusion MRI with long acquisition periods in fixed whole brain preparations. This approach allowed high resolution images which can reveal submillimetric structures. Such high quality would be difficult to achieve *in vivo* due to motion artifacts and limited acquisition times. Moreover, the use of high angular resolution schemes provides more accurate diffusion related parameters even using diffusion tensor imaging (DTI) approaches [36]. Since segmentation of anatomic regions in small developing brains presents substantial challenges [37], we explored a voxel based analysis (VBA) approach in order to overcome the limitations described for manual delineation. VBA approach performs the analysis of the whole brain voxel-wise and identifies anatomical areas presenting differences avoiding the need of *a priori* hypothesis or previous delineation [38]. The aims of the study were to describe the anatomical pattern of fetal brain maturation changes as assessed by MRI, and to establish functional-structural correlates of fetal brain reorganization induced by IUGR.

## Materials and Methods

**Figure 1:**
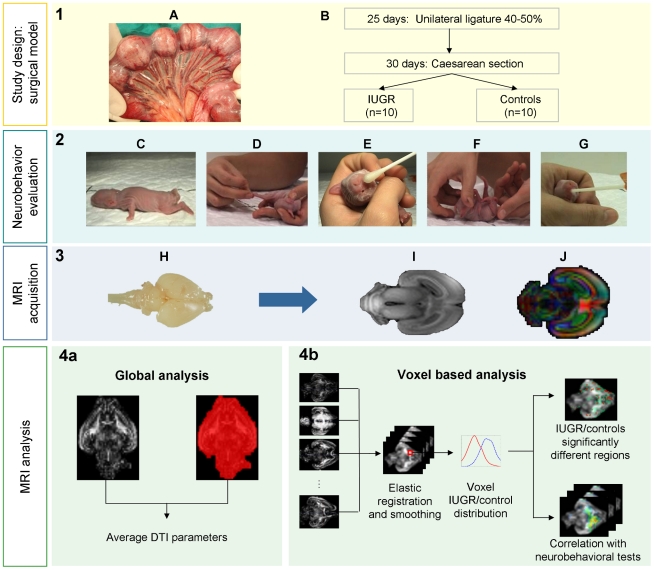
Schematic and graphical representation of study design and methods. PANEL 1: (A) Illustrative image of unilateral ligation of 40–50% of uteroplacental vessels at 25 days of pregnancy, (B) Scheme of surgical procedures and study groups. PANEL 2: Illustrative pictures of neurobehavioral evaluation of locomotion (C), tone (D), smelling test (E), righting reflex (F) and sucking and swallowing (G) performed at +1 postnatal day. PANEL 3: MRI acquisition. Fixed brains (H) were scanned to obtain a high resolution T1 weighted (I) images and diffusion weighted images (J). PANEL 4a: MRI global analysis. After masking brain volume, global analysis was performed to obtain average DTI parameters (FA, ADC, radial diffusivity, axial diffusivity, linearity, planarity and sphericity). PANEL 4b: Voxel based analysis was performed by elastic registration to a reference FA map. Once subject brains were registered and smoothed, diffusion-related parameters values distribution for each voxel was analyzed to identify areas with statistically significant different distribution in IUGR and the correlation of changes with neurobehavioral tests.

The methodology of the study is shown in Figure 1. Each of the steps of the procedure is detailed in this section.

### 1. Study protocol and procedures

#### a) Animals and study protocol

Animal experimentation of this study was approved by the Animal Experimental Ethics Committee of the University of Barcelona (permit number: 206/10-5440). Animal handling and all the procedures were performed following all applicable regulations and guidelines of the Animal Experimental Ethics Committee of the University of Barcelona. The study groups were composed by 10 cases with induced IUGR and 10 sham controls obtained from New Zealand pregnant rabbits provided by a certified breeder. Dams were housed for 1 week before surgery in separate cages on a reversed 12/12 h light cycle, with free access to water and standard chow.

At 25 days of gestation (term at 31 days), we performed ligation of 40–50% of uteroplacental vessels following a previously described technique [32] and cesarean section was performed at 30 days of gestation. At postnatal day +1, neurobehavioral evaluation was performed and afterwards neonates were sacrificed. Then, brains were collected and fixed with 4% paraformaldehyde phosphate-buffered saline (PBS).

#### b) Surgical model

Induction of IUGR was performed at 25 days of gestation as previously described [32]. Briefly, after tocolysis and antibiotic prophylaxis administration, an abdominal midline laparotomy was performed under anaesthetic condition. Gestational sacs of both horns were identified and, in one uterine horn, 40–50% of the uteroplacental vessels of all gestational sacs were ligated. After the procedure the abdomen was closed in two layers with a single suture of silk (3/0). Postoperative analgesia was administered and animals were again housed with free access to water and standard chow for 5 days until delivery and well-being was controlled each day.

Cesarean section was performed at 30 days of gestation and living and stillborn fetuses were obtained. After delivery, all living newborns were weighed and identified by ear punching.

#### c) Neurobehavioral test

Neurobehavioral evaluation was performed at postnatal day +1 following methodology previous described by Derrick et al. [35]. For each animal, the testing was videotaped and scored on a scale of 0–3 (0, worst; 3, best) by a blinded observer. Locomotion on a flat surface was assessed by grading the amount of spontaneous movement of the head, trunk, and limbs. Tone was assessed by active flexion and extension of the forelimbs and hindlimbs (0: No increase in tone, 1: Slight increase in tone when limb is moved, 2: Marked increase in tone but limb is easily flexed, 3: Increase in tone, difficult passive movement, 4: Limb rigid in flexion or extension). The righting reflex was assessed when the pups were placed on their backs and the number of times turned prone from supine position in 10 tries was registered. Suck and swallow were assessed by introduction of formula (Lactadiet with omega 3; Royal Animal, S.C.P.) into the pup's mouth with a plastic pipette. Olfaction was tested by recording time to aversive response to a cotton swab soaked with pure ethanol. After neurobehavioral evaluation, neonates were sacrificed by decapitation after administration of Ketamine 35 mg/kg given intramuscularly. Brains were collected and fixed with 4% paraformaldehyde phosphate-buffered saline (PBS), for 24 hours at 4°C.

#### d) Magnetic resonance acquisition

MRI was performed on fixed brains using a 7T animal MRI scanner (Bruker BioSpin MRI GMBH). High-resolution three-dimensional T1 weighted images were obtained by a Modified Driven Equilibrium Fourier Transform (MDEFT) 3D sequence with the following parameters: echo time (TE) = 3.5 ms, repetition time (TR) = 4000 ms, slice thickness = 0.25 mm with no interslice gap, 84 coronal slices, in-plane acquisition matrix of 128×128 and Field of View (FoV) of 32×32 mm^2^, which resulted in a voxel dimension of 0.25×0.25×0.25 mm^3^. Diffusion weighted images (DWI) were acquired by using a standard diffusion sequence covering 126 gradient directions with a b-value of 3000 s/mm^2^ together with a reference (b = 0) image. Other experimental parameters were: TE = 26 ms, TR = 250 ms, slice thickness = 0.35 mm with no interslice gap, 60 coronal slices, in-plane acquisition matrix of 46×46 and FoV of 16×16 mm^2^, which resulted in a voxel dimension of 0.35×0.35×0.35 mm^3^. Total scan time for both acquisitions was 14 h 20 m 04 s.

### 2. MRI processing and analysis

#### a) Processing of diffusion MRI

As a first step, the brain was segmented from the background by means of customized software implemented in Matlab 2011a (The Mathworks Inc, Natick, MA, USA). In brief, the 126 DWI images were averaged to generate a high SNR isotropic diffusion weighted image (iDWI) that was used to create a binary mask to segment the brain from the background, in a similar way as previously described [39]. In brief, iDWI of each subject was min-max normalized, and non-brain tissue values were estimated to have values below 5% of the maximum of the iDWI normalized volume. After applying the threshold, internal holes in the mask were filled by 3D morphological closing and isolated islands were removed by 3D morphological opening. This mask was used to estimate brain volume and constrain the area where the diffusion related measures were analyzed.

Tensor model of diffusion MRI was constructed by using MedINRIA 1.9.4 [40] (available at www-sop.inria.fr/asclepios/software/MedINRIA/). Once the tensors were estimated at each voxel inside the brain mask, a set of measures describing the diffusion were computed: apparent diffusion coefficient (ADC), fractional anisotropy (FA), axial and radial diffusivity and the coefficients of linearity, planarity and sphericity [24], [41]. They are all based on the three eigenvalues of each voxel tensor (λ_1_, λ_2_, λ_3_). ADC measures the global amount of diffusion at each voxel, whereas axial diffusivity measures the diffusion along the axial direction, that is, along the fiber direction. On the other hand, radial diffusivity provides information of the amount of diffusion orthogonal to the fiber direction. The other parameters are related to the shape and anisotropy of the diffusion. FA describes the anisotropy of the diffusion, since diffusion in fibers is highly anisotropic its value is higher in areas where fiber bundles are [24]. Linearity, planarity and sphericity coefficients describe the shape of the diffusion; higher values of the linear coefficient indicates that diffusion occurs mainly in one direction; higher planarity involves that diffusion is performed mostly in one plane, and higher values of sphericity are related to isotropic diffusion [41].

#### b) Global analysis

The parameters described in the previous section were computed at each voxel belonging to the brain mask, and their value was averaged in the whole brain, in order to perform a global analysis of the differences between controls and IUGR.

In addition, so as to avoid potential confounding values produced by GM and cerebrospinal fluid (CSF), a second mask was applied to analyse the changes in the WM. It is known that WM is related to higher values of FA, and therefore a FA threshold can be defined to identify this kind of tissue. Thus, masks were built by a set of thresholds ranging from 0.05 to 0.40, and the diffusion parameters inside these masks were computed. The consistency of the results achieved using the set of masks was analyzed (Fig. S1A). By visual inspection, it was estimated that a threshold of FA = 0.20 rendered the best discrimination of structures of WM in the brains (Fig. S1B), and thus, this threshold was used in further analyses.

#### c) Regional analysis: Manual delineation

**Figure 2:**
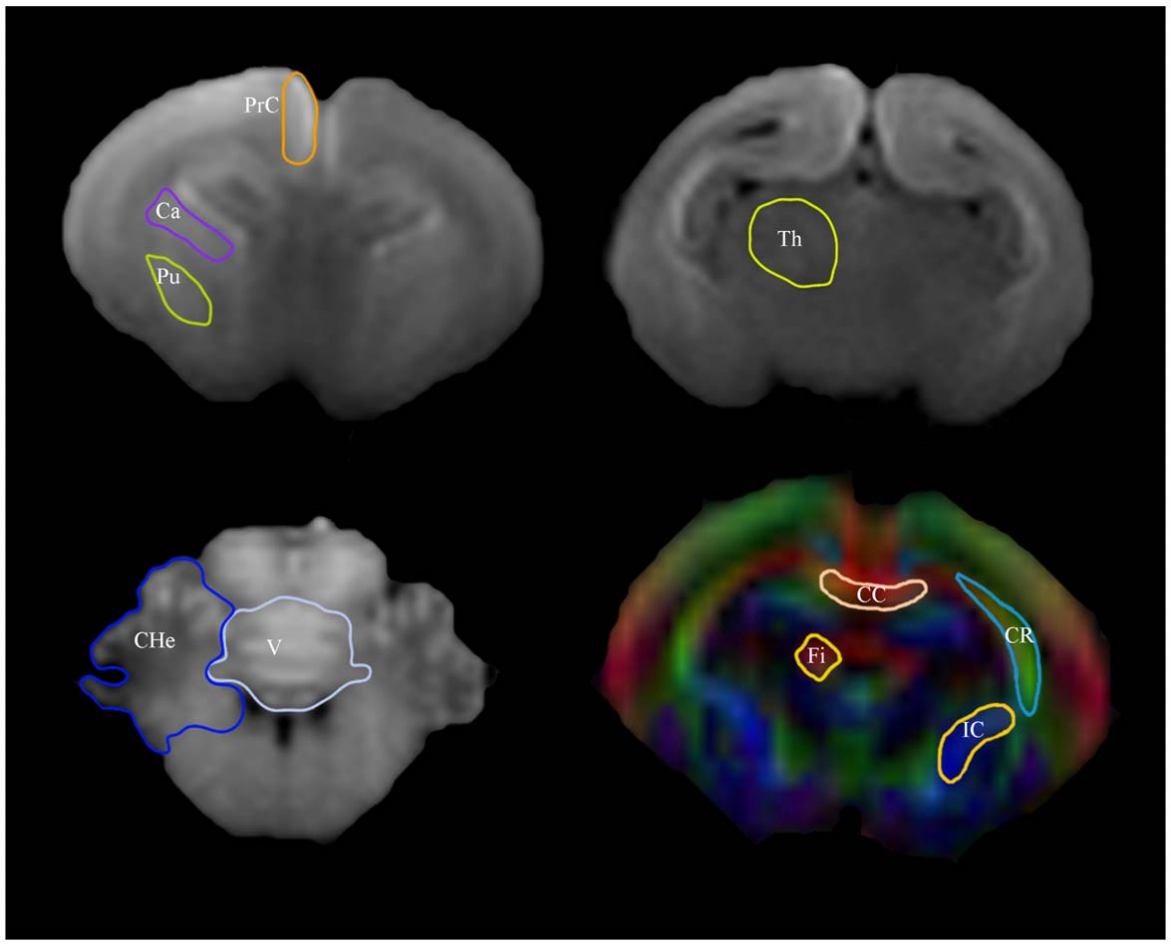
Manual delineation of regions of interest (ROIs). Coronal slices with manual delineation of ROIs. GM structures were delineated in T1 weighted images including prefrontal cortex (PrC), caudate nucleus (Ca), putamen (Pu), thalamus (Th), cerebellar hemisphere (CHe), and vermis (V). WM structures were delineated directly in FA map including corpus callosum (CC), corona radiata (CR); internal capsule (IC), and fimbria hippocampus (Fi).

Manual delineation of GM regions of interest (ROIs) was performed in T1 weighted images including thalamus, putamen, caudate nucleus, prefrontal cortex, cerebellar hemispheres and vermis (Fig. 2). WM ROIs (corpus callosum, fimbria of hippocampus, internal capsule and corona radiata) were delineated directly in FA map (Fig. 2).

GM ROIs were co-registered to DWI by applying a previously calculated affine transformation of the T1 weighted images to DWI space. Mean diffusion related measures were obtained including ADC, axial and radial diffusivities, FA, linearity, planarity and sphericity coefficients.

#### d) Regional analysis: Voxel based analysis

All rabbit brains were registered to a reference brain using their FA volumes [42] by means of an affine registration that maximized mutual information of volume [43] followed by an elastic warping based on diffeomorphic demons [44] both available in MedINRIA 1.9.4 software. Registered volumes were smoothed with a Gaussian kernel of 3×3×3 voxels (1.05×1.05×1.05 mm^3^) with standard deviation of one voxel (0.35 mm) in order to compensate for possible misregistrations, reduce noise and signal variations and reduce the effective number of multiple comparisons in the statistical testing thus improving statistical power [45].

Once the images are aligned to the reference, it can be assumed that the voxels in the same location in all the registered images belong to the same structure, and therefore, they can be compared. Voxel-wise t-test was performed, obtaining the voxels with a statistically significant different distribution of diffusion related parameters between controls and IUGR. Moreover, in this study, the Spearman correlation between the diffusion parameters and the neurobehavior test outcome at each voxel was also computed, to identify which regions were related to the observed changes in neurobehavioral tests.

Since VBA requires the definition of a reference brain, results could be biased by this choice. In order to avoid such a bias and increase the reliability of the obtained results, the VBA procedure was repeated taking all subjects as template, and only the regions where differences appeared consistently among the different templates were considered. In this way, the variability produced by the arbitrarily choice of the reference template is discarded.

### 3. Statistical analysis

Given the absence of preliminary data and the difficulty in estimating the magnitude of differences, sample size was arbitrarily established at 10 subjects and 10 controls. For quantitative variables, normality was assessed by the Shapiro-Francia W′ test [46]. Normal-distributed quantitative variables were analysed by t-test. Non-normal distributed variables were analysed with the non-parametric Mann–Whitney U test. Correlation between different variables was assessed by means of Spearman correlation. In VBA approach, registered and smoothed volumes of FA, ADC, radial and axial diffusivity and linearity, planarity and sphericity coefficients were used to obtain volumetric maps of t-statistics, showing the voxels that presented a significant difference between groups (uncorrected p<0.01 and p<0.05). In addition, a correlation volume (ρ) was also calculated for each neurofunctional item, expressing positive and negative Spearman correlations between FA and neurofunctional outcome. Image analysis and processing was performed by means of customized software implemented in Matlab 2011a (The Mathworks Inc, Natick, MA, USA). SPSS 15.0 (SPSS Inc., Chicago, IL, USA) was used for statistical analysis.

## Results

### 1. Perinatal data and neonatal neurobehavior

Birth weight was significantly lower in cases than in controls (controls vs. cases: 47.0±9.3 g. vs. 30.4±12.2 g., p = 0.007). Regarding neurobehavioral test, growth restricted pups showed poorer results in all parameters, reaching significance in righting reflex, tone of the limbs, locomotion, lineal movement, fore-hindpaw distance, head turn during feeding and smelling response (Table 1 and Video S1).

**Table 1:** Neurobehavioral test results in study groups.

	*Control n = 10*	*IUGR n = 10*	*p*
Posture, score*	3.0 (0)	3.0 (1)	0.143
Righting reflex, number of turns	8.7 (1.5)	6.3 (3.0)	0.035
Tone, score*	0 (0)	1.0 (1.5)	0.019
Locomotion, score*	3.0 (0)	2.0 (2)	0.005
Circular motion, score*	2.0 (1)	2.0 (1)	0.247
Intensity, score*	3.0 (0)	2.5 (2)	0.089
Duration, score*	2.0 (0)	1.5 (1)	0.052
Lineal movement, line crosses in 60 sec	2.8 (1.4)	1.1 (1.1)	0.009
Fore–hindpaw distance, mm †	0.7 (1.9)	7.6 (5.4)	0.007
Sucking and swallowing, score*	3.0 (1)	1 (2)	0.075
Head turn, score*	3.0 (1)	2.0 (1)	0.043
Smelling test, score * †	3.0 (1)	1.0 (0)	0.006
Smelling test time, sec †	4.0 (1)	8.5 (5)	0.021

IUGR: intrauterine growth restriction; sec: seconds; mm: millimeters.

Values are mean and standard deviation (mean (sd)) or median and interquartile range (median (IQ)) when appropriate.

*U Mann-Whitney.

† Data available for 7 controls and 8 cases.

### 2. Brain MRI analysis

MRI analysis revealed significant lower brain volume in growth restricted group (controls vs. cases: 1345±110 mm^3^ vs. 1211±152 mm^3^, p = 0.037). When brain volume was adjusted by means of the brain volume/birth weight ratio, case group showed significantly higher values (controls vs. cases: 29.2±7.9 vs. 39.9±6.5, p = 0.033).

#### a) Global analysis

Table 2 depicts the results of global analysis of diffusion related parameters. Whole brain analysis revealed non-significantly higher ADC values and significantly lower FA and linearity values in the growth restricted group. When the WM mask was applied, FA significantly differed between cases and controls (Fig. S1). Regarding the correlation between neurobehavioral and diffusion parameters, head turn during feeding was significantly positively correlated with global FA (r = 0.489, p = 0.034), maintained when the WM mask was applied (r = 0.652, p = 0.003) (Table 3). Similarly, locomotion was significantly negatively correlated with global ADC (r = −0.459, p = 0.048) and radial diffusivity (r = −0.493, p = 0.032) when WM mask was applied (Table 3)

**Table 2:** Whole brain and white matter global analysis of diffusion parameters in study groups.

	*Control n = 10*	*IUGR n = 10*	*p*
***Whole brain***			
Fractional anisotropy	0.16 (0.02)	0.15 (0.02)	0.048
Apparent Diffusion Coefficient (×10^−3^ mm^2^/s)*	0.44 (0.08)	0.47 (0.10)	0.353
Axial diffusivity (×10^−3^ mm^2^/s)*	0.52 (0.10)	0.54 (0.11)	0.393
Radial diffusivity (×10^−3^ mm^2^/s)*	0.41 (0.07)	0.43 (0.10)	0.393
Sphericity coefficient	0.74 (0.02)	0.76 (0.02)	0.061
Linearity coefficient	0.16 (0.02)	0.15 (0.02)	0.044
Planarity coefficient	0.10 (0.01)	0.10 (0.01)	0.368
***White matter (threshold FA>0.2)***			
Fractional anisotropy	0.27 (0.01)	0.26(0.00)	0.019
Apparent Diffusion Coefficient (×10^−3^ mm^2^/s)*	0.42(0.08)	0.44(0.11)	0.353
Axial diffusivity (×10^−3^ mm^2^/s)*	0.55 (0.10)	0.58 (0.15)	0.393
Radial diffusivity (×10^−3^ mm^2^/s)*	0.36 (0.06)	0.38 (0.09)	0.247
Sphericity coefficient	0.60 (0.02)	0.61 (0.01)	0.033
Linearity coefficient	0.29 (0.02)	0.28 (0.02)	0.201
Planarity coefficient	0.11 (0.02)	0.11 (0.03)	0.877

Values are mean and standard deviation (mean (sd)) or median and interquartile range (median (IQ)) when appropriate.

*U Mann-Whitney.

**Table 3:** Mean correlation coefficients between diffusion parameters and neurobehavioral test results (Spearman's correlation).

	FA	ADC	Axial D	Radial D	FA (FA>0.2)	ADC (FA>0.2)	Axial D (FA>0.2)	Radial D (FA>0.2)
Posture	0.283	−0.024	−0.047	−0,047	0,283	−0.071	−0.071	−0.094
Righting reflex	0.016	−0.140	−0.144	−0.132	0.082	−0.151	−0.154	−0.157
Tone	−0.045	0.219	0.208	0.191	−0.341	0.201	0.201	0.243
Locomotion	0.168	−0.451	−0.434	−0.409	0.283	**−0.459** *	−0.452	**−0.493** *
Circular motion	0.402	−0.148	−0.046	−0.178	0.247	−0.121	−0.135	−0.150
Intensity	0.253	−0.230	−0.190	−0.206	0.356	−0.190	−0.174	−0.230
Duration	−0.130	−0.274	−0.336	−0.238	0.156	−0.310	−0.285	−0.326
Lineal movement	−0.009	0.169	0.104	0.163	0.177	0.132	0.111	0.125
Fore–hindpaw distance	−0.361	0.309	0.295	0.302	−0.508	0.331	0.312	0.394
Sucking and swallowing	0.122	−0.005	−0.036	−0.020	0.434	−0.046	−0.036	−0.077
Head turn	**0.489** *	−0.118	0.030	−0.163	**0.652** **	−0.030	0.015	−0.089
Smelling test	0.238	−0.479	−0.423	−0.483	0.335	−0.423	−0.400	−0.460
Smelling test time	−0.166	0.281	0.226	0.277	−0.368	0.190	0.151	0.228

FA: Fractional anisotropy, ADC: Apparent Diffusion Coefficient, Axial D: Axial Diffusivity, Radial D: Radial Diffusivity.

*p<0.05,

**p<0.001.

#### b) Regional analysis: Manual delineation

ROIs analysis of diffusion parameters only found differences in right fimbria of hippocampus, showing decreased values of FA in IUGR (p = 0.048) (Table S1).

#### c) Regional analysis: Voxel based analysis

**Figure 3:**
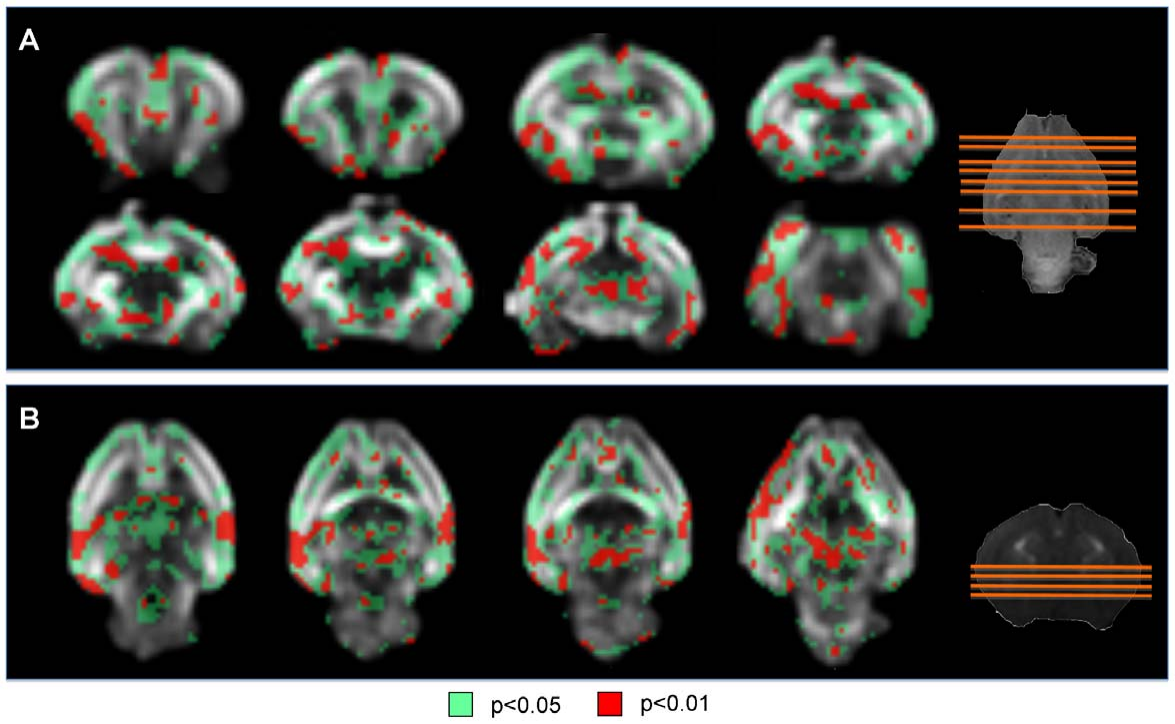
Fractional anisotropy values: regions showing statistically significant differences between cases and controls. Slices of the smoothed reference FA image. Red areas have a significance of p<0.01, green areas have a significance of p<0.05. The slices displayed contain representative anatomical structures. Slice locations are shown in the T1 weighted images in the right. (A) Coronal slices from anterior to posterior. (B) Axial slices from superior to inferior.

**Figure 4:**
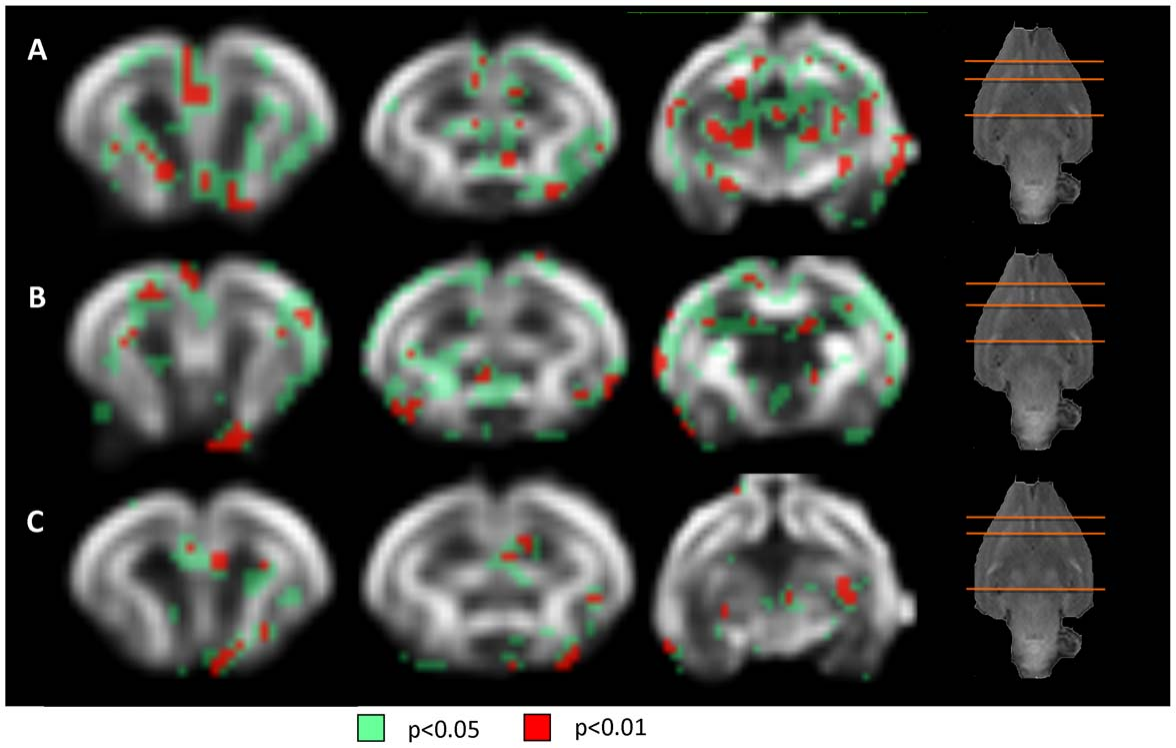
Linearity, planarity and sphericity coefficients: regions showing statistically significant differences between cases and controls. Coronal slices of the smoothed reference FA image. Red areas have a significance of p<0.01, green areas have a significance of p<0.05. Slice locations are shown in the T1 weighted images in the right. The slices displayed representative anatomical regions showing increased sphericity coefficient (A) and decreased linearity (B) and planarity (C) coefficient.

When VBA analysis was applied, statistically significant differences were found in FA distribution between cases and controls in multiple structures such as different cortical regions (frontal, insular, occipital and temporal), hippocampus, putamen, thalamus, claustrum, medial septal nucleus, anterior commissure, internal capsule, fimbria of hippocampus, medial lemniscus and olfactory tract (Fig. 3). Significant differences were also found in the distribution of the coefficients of linearity (decreased), planarity (decreased), and sphericity (increased), in the same regions showing changes in FA distribution (Fig. 4). In addition, significantly decreased planarity and increased sphericity were also observed in corpus callosum. In addition, there were very few and randomly distributed spots showing statistically significant changes in ADC and radial and axial diffusivity.

### 3. Correlation between MRI diffusion and neurobehavioral outcome

**Figure 5:**
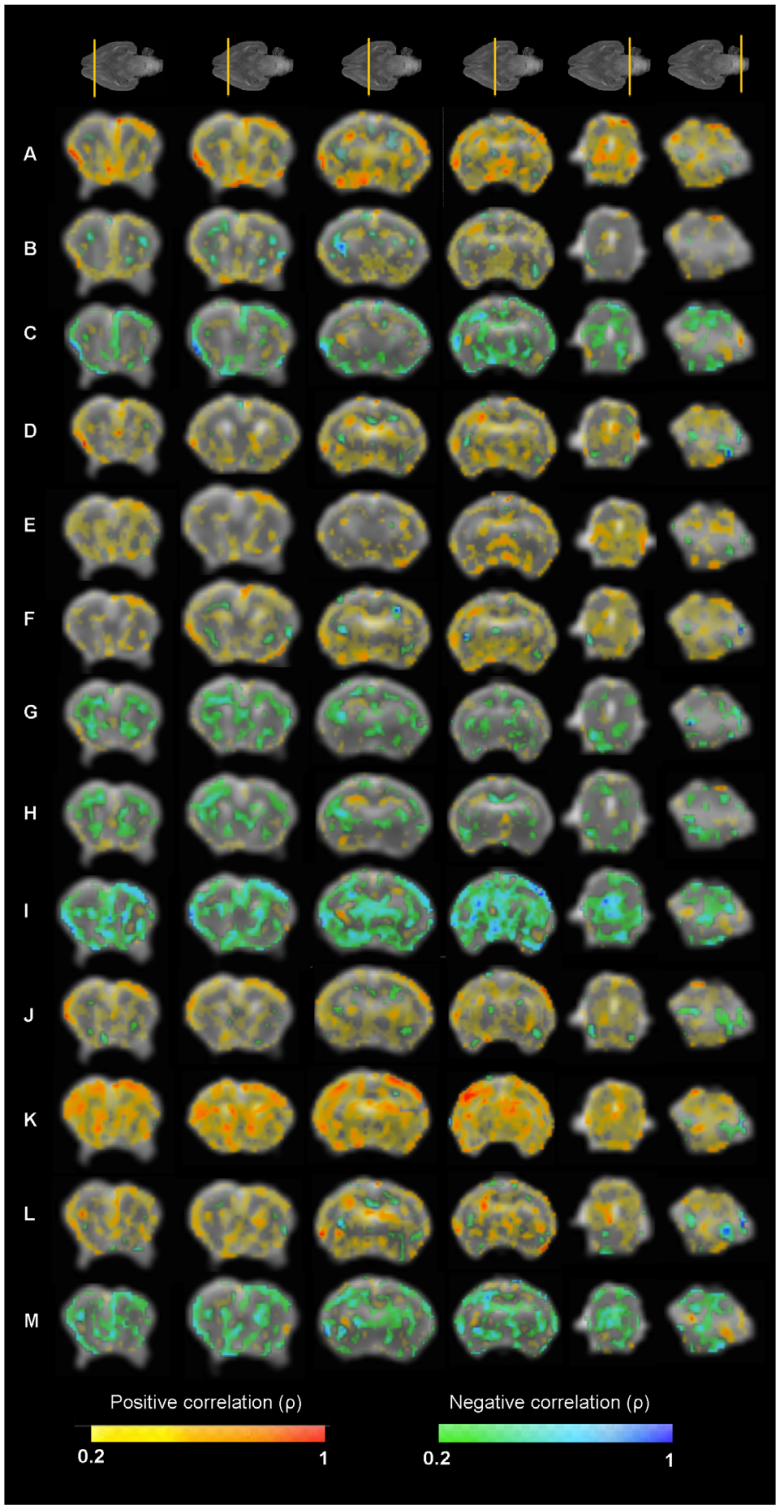
Correlation maps between neurobehavioral test items and fractional anisotropy values. Coronal slices (from anterior to posterior) of the smoothed reference FA image. Colormap highlights the areas where the correlation coefficient is higher than 0.2. (A) Posture, (B) Righting reflex, (C) Tone, (D) Locomotion, (E) Circular motion, (F) Intensity, (G) Duration, (H) Lineal movement, (I) Fore-hindpaw distance, (J) Sucking and swallowing, (K) Head turn, (L) Smelling test, (M) Smelling test time.

FA map showed multiple areas correlated with most of neurobehavioral domains, being posture, locomotion, circular motion, intensity, fore-hindpaw distance and head turn the domains showing more statistically significant correlated areas (Fig. 5, Table 4 and Table S2). Cortical and subcortical GM areas were mainly correlated with posture, locomotion and head turn; and WM structures essentially with posture, locomotion, sucking and swallowing and head turn parameters. Interestingly, hippocampus is the GM structure that presented more correlations with neurobehavioral domains (locomotion, circular motion, lineal movement, fore-hindpaw distance and head turn). Within WM structures, both anterior commissure and fimbria of hippocampus were the areas correlated with a bigger amount of neurobehavioral items. To be highlighted, olfactory items correlate with very specific areas, including prefrontal and temporal cortex, caudate nucleus and olfactory tract.

**Table 4:** Significant correlations (p<0.01) between neurobehavioral domains and fractional anisotropy in brain regions.

	Positive correlation	Negative correlation
Posture	Occipital cortex, temporal cortex, thalamus, anterior commissure, olfactory tract	-
Righting reflex	Occipital cortex, temporal cortex	-
Tone	-	-
Locomotion	Hippocampus, insular cortex, frontal cortex, occipital cortex, temporal cortex	-
Circular motion	Hippocampus, frontal cortex, occipital cortex, temporal cortex, thalamus	-
Intensity	Hippocampus, temporal cortex, claustrum, olfactory tract, optic tract	-
Duration	-	-
Lineal movement	-	-
Fore–hindpaw distance	-	Hippocampus, Frontal cortex, occipital cortex, temporal cortex, thalamus, fimbria of hippocampus
Sucking and swallowing	Frontal cortex, occipital cortex, temporal cortex	-
Head turn	Hippocampus, frontal cortex, occipital cortex, temporal cortex, thalamus, anterior commissure, corona radiata, internal capsule.	-
Smelling test	Prefrontal cortex, temporal cortex Olfactory tract,	-
Smelling test time	-	Temporal cortex

## Discussion

In this study we developed a rabbit model to evaluate functional and structural impact of IUGR, providing high-resolution MRI description of the anatomical patterns of brain maturational changes occurring *in utero*. We demonstrated that IUGR was associated with different patterns of brain diffusivity in multiple brain regions, which were significantly correlated with the neurobehavioral impairments observed. The model developed may be a powerful tool to correlate functional and structural brain information with histological, molecular and other imaging techniques. In addition, it allows detailed regional assessment of the impact of interventions in the complex patterns of brain reorganization induced by adverse prenatal environment.

### Neonatal neurobehavior

It is known that IUGR in humans is associated with neonatal neurodevelopmental dysfunctions [4], [5], being attention, habituation, regulation of state, motor and social-interactive clusters the most affected [5]. In a similar manner, growth restricted rabbit pups in this model showed weakened motor activity and olfactory function, which is their principal way of social interactions [47]. The findings reinforce previous evidence suggesting the capability of this animal model to reproduce features of human IUGR [32], [33]. Previous studies suggested the ability of the rabbit model to illustrate the neonatal effects of acute severe prenatal conditions. Thus, hypoxic-ischemic injury and endotoxin exposure produce hypertonic motor deficits [35], [48], reduced limb movement [49] and olfactory deficits [50] in this model. The present study demonstrates that selective ligature of uteroplacental vessels is suitable to reflect the neurodevelopmental impact of mild and sustained reduction of placental blood flow occurring in IUGR. These results illustrate a more general concept that lower animal species are also susceptible of developing brain reorganization *in utero*, and therefore they are suitable models to assess the chronic effects of adverse intrauterine environment on brain development.

### MRI global analysis

Changes in brain diffusivity and anisotropy have previously been reported after acute severe hypoxic experimental conditions in adults [26] and developing brain [27]. Placental insufficiency results in mild and sustained injury, which may challenge the ability to find obvious differences between groups. With the purpose of detecting subtle changes we used high-resolution MRI acquisition in fixed whole brain preparations. This approach allows revealing submillimetric tissue structure differences, particularly in the GM, which are difficult to detect *in vivo* [21]. As a trade-off, fixation process may decrease brain water content reducing ADC absolute values, although diffusion anisotropy is preserved [51].

In growth restricted pups, global decreased FA values were demonstrated in both whole brain and WM mask analysis. Findings are similar to those observed in acute hypoxic-ischemic injury models [52] and perinatal asphyxia in humans [23] demonstrating decreased values in FA particularly in WM areas. Aside from acute models, preliminary evidence in neonates with cyanotic congenital heart defects suggests also the presence of brain FA changes [53], [54]. FA indicates the degree of anisotropic diffusion and typically increases in WM areas during brain maturation, being closely related with myelination processes [23]. After acute hypoxic-ischemic injury in rat pups, decreased values of FA have been related with decreased myelin content in WM areas [55]. However, we acknowledge that there is a chance that decreased FA could also be explained by increases in crossing fibers [56]. Consistently with decreased FA, the findings demonstrated that IUGR had a significant increase in sphericity, changes that have been related with reduced organization of WM tracts [41]. Therefore, the results of the study are consistent with the presence of decreased WM myelination and brain reorganization after exposure to IUGR in the rabbit model.

Global diffusivity analysis revealed a non-significant trend for increased ADC in the IUGR group. ADC is directly related with the overall magnitude of water diffusion, typically decreasing as brain maturation occurs [23]. In addition, after perinatal acute hypoxic-ischemic event, it shows a dynamic process with a quickly decrease followed by a pseudo-normalization to finally increase to higher values than normal [27]. In humans, ADC values have been demonstrated to be increased in multiple brain regions after chronic fetal conditions including IUGR [30] and fetal cardiac defects [29], [53], [54]. In addition, increased ADC values have been reported after prenatal acute hypoxic-ischemic injury in hypertonic rabbits [52]. We found a non-significant trend to increased ADC values in IUGR. We acknowledge that sample size may have prevented to detect subtle differences in ADC. In any event, the lack of remarkable differences in ADC is possibly a reflection of the abovementioned notion that IUGR results in delayed brain maturation and reorganization rather than in significant brain injury [22], [57]. Further histological studies may help to clarify essential information about microstructural changes and allow correlations with findings in diffusion parameters here reported.

The finding of a significant decrease in global and regional analyses of FA together with the lack of changes in ADC could seem inconsistent. However, previous evidence indicates that both parameters are actually independent [23]. It is known that the FA increase takes place before the histologic appearance of myelin [58]–[60]. In rabbits, oligodendrocyte proliferation and maturation occurs from 29 days of pregnancy to postnatal day +5, with myelination starting around postnatal day +3 [48]. Thus, increases in FA in the “premyelinating state” could be due to other factors, including an increase in the number of microtubule-associated proteins in axons, changes in axon calibre, and the rapid increase in the number of oligodendrocytes [60]. On the other hand, the ADC decrease during bran maturation is not fully understood. It has been postulated to be due to the concomitant decrease in overall water content [59]. Thus, we hypothesize that the pattern of changes described in our model with significantly decreased FA and lack of marked changes in ADC could be explained by two mechanisms. First, rabbit pups suffering IUGR have histological changes in brain organization during the “premyelinating state”, which would lead to the decrease in FA values. Secondly, as myelin has not appeared in postnatal day +1 (neither in cases nor in controls), water content and the restriction to its movement which conditions ADC values remain similar in both groups. We acknowledge however that the results in ADC could have also been influenced by fixation processes used in this study, which decrease water content in a non-homogeneous, and therefore non-predictable manner [51].

### MRI regional analysis

Regional analysis of diffusivity parameters may provide information of the anatomical pattern of brain microstructural changes in IUGR. As expected, manual brain segmentation showed limited results and significant differences in a few brain areas. As shown in previous studies, this approach has limitations in small structures, due to the difficulty in obtaining accurate delineations [61] and to the partial volume effects [37]. Since these limitations were known, a VBA strategy was applied. VBA approach performs the analysis of the whole brain voxel-wise avoiding the need of *a priori* hypothesis or previous delineation [38], and allowed to localize regional differences between cases and controls in FA distribution.

Cortical and subcortical GM areas were the most altered regions and, as expected, regional reductions in FA showed significant high correlations with functional impairment. Cortical changes are a feature of IUGR, as suggested by decreased cortical volume [13] and discordant patterns of gyrification due to pronounced reduction in cortical expansion in neonates [15] and differences in GM brain structure in infants [16] suffering this condition. Our results support the notion that these changes are based on microstructural differences. In line with this contention, microstructural changes in cortical regions have previously been demonstrated in a sheep model of IUGR, including cortical astrogliosis, fragmentation of fibers and thinner subcortical myelin sheaths [62]. Importantly, these histological features have been shown to correlate with decreased FA in cortex [28] and subcortical WM [63]. Regional analysis demonstrated that among GM affected regions, the hippocampus showed the highest number of significant correlations with neurobehavioral domains. The hippocampus is known for its crucial role in cognitive function such as memory and learning. In human IUGR neonates, a reduction in neonatal hippocampal volume was associated with poor neurofunctional outcomes in neonatal period including autonomic motor state, attention-interaction, self-regulation and examiner facilitation [14]. Additionally, previous experimental data have demonstrated reduced number of neurons in hippocampus [64] and alterations in the dendritic morphology of pyramidal neurons [65] after IUGR. In summary, the findings support that impaired neurocognition in IUGR is mediated by microstructural changes in cortical and subcortical areas detectable with diffusion MRI, with hippocampus playing an important role.

Regional analysis revealed changes in multiple WM structures. The most pronounced differences were found in the internal capsule, anterior commissure and fimbria of hippocampus, which showed correlations with locomotion parameters, posture, sucking and swallowing and head turn. Changes in WM structures have also been reported in human fetuses, with increased ADC in pyramidal tract in IUGR [30] and increased ADC in multiple WM areas in fetuses [29] and newborns [53], [54] with congenital cardiac defects. Consistently with our results, prenatal chronic hypoxia models have demonstrated inflammatory microgliosis, mild astrogliosis [66], and a delay in the maturation of oligodentrocytes leading to a transient delay in myelination [57]. These changes result in global reduction in axonal myelination in absence of overt WM damage [67] which in turn is reflected by decreased values of FA [63] as observed in this study. Interestingly, anterior commissure and fimbria of hippocampus, which showed significant differences in FA distribution demonstrated by VBA, were the WM structures correlated with more altered neurobehavioral items, especially posture, reflex responses and locomotion. Of note, these two WM tracts connect GM structures that also presented significantly decreased FA demonstrated by VBA. Anterior commissure contains axonal tracts connecting temporal lobes and fimbria of hippocampus contains efferent fibers from hippocampus. In addition, changes in olfactory tract, which is closely related with olfaction, were significantly correlated with smelling test results. This finding was consistent with previous data demonstrating that neurons of the olfactory epithelium in rabbit are sensitive to global acute hypoxia-ischemia [50]. Finally, regional analysis of planarity, linearity and sphericity coefficient revealed significant differences with decreased values of linearity and planarity and increased sphericity in the same regions with decreased FA in IUGR. These findings suggest the presence of altered and delayed WM organization and maturation and does not support that FA decrease be due to an increase in crossing fibers [41]. In summary, this study characterized regional alterations in WM diffusion parameters, findings which were in line with GM data and further suggest the presence of microstructural regional changes underlying brain reorganization in IUGR. Furthermore, reduced WM FA could indicate connectivity changes and a role for MRI diffusion connectomics for the development of more robust biomarkers of brain injury in IUGR, which deserve investigation in future studies.

### Strengths and limitations

Some issues must be noted concerning the methodology followed. Firstly, the absolute values of ADC obtained in this study were lower than those previously reported in neonatal rabbit brain [52], [68]. As abovementioned, that could be explained by the fact that brain fixation decreases water content in the brain reducing ADC values [51]. However, in order to preserve diffusion contrast we used high b-values as previously suggested [69]. In addition, all the brains followed the same fixation process and, theoretically, must be affected in a similar way. Secondly, in the global analysis, a FA thresholding approach was used to identify the voxels belonging to the WM. Although this thresholding has usually been described in order to segment the WM in human brains [70], to the best of our knowledge, it has not been defined for perinatal rabbit brain. Therefore, different thresholds were analyzed, showing that the differences between controls and IUGR are preserved for a wide range of values of the FA threshold (Fig. S1 and Fig. S2). Thirdly, regional analysis of the images has been performed by means of VBA technique in order to overcome manual delineation limitations. However, the use of VBA implies weaker statistical power due to the large number of voxels tested [45], increasing type I error rate even after smoothing diffusion related measures volumetric maps. Another issue concerning VBA is that the method requires registration of all the subjects in the dataset to a template volume, and therefore the arbitrary choice of this template could bias the result [45]. As described in the methodology section, this issue has been addressed by repeating the VBA considering each of the subjects as the reference, ensuring the consistency of the regional changes identified. Finally, this work is based on diffusion related parameters, which measure either the amount of diffusivity or the anisotropy of the diffusion, but do not provide information about diffusion direction and therefore, about the fiber bundles trajectories. Further connectivity studies, where WM tracts connecting different areas are identified, will permit a better understanding of the consequences of IUGR in the brain development.

### Conclusions

In conclusion, we developed a fetal rabbit model reproducing neurobehavioral and neurostructural consequences of IUGR. Diffusion MRI in whole organ preparations allowed showing differences on global and regional diffusion related parameters, revealing in detail the pattern of brain microstructural changes produced by IUGR already at birth and their functional correlates in early neonatal life. The results illustrate that sustained intrauterine restriction of oxygen and nutrients induces a complex pattern of maturational changes, in both GM and WM areas. The model here described permitted to characterize the most significantly affected regions. These anatomical findings could be of help in multi-scale studies to advance in the understanding of the mechanisms underlying abnormal neurodevelopment of prenatal origin. In addition, MRI diffusion changes can be used to monitor the impact of interventions. WM changes warrant the development of further studies for the development of imaging biomarkers of brain reorganization in IUGR and other fetal chronic conditions.

## Supporting Information

Figure S1 **Fractional anisotropy thresholds in the global analysis.** (A) Control and IUGR group distribution of average FA on the mask of WM computed with different FA thresholds. Error bars depict ±1 standard deviation. (B) Representative axial and coronal slices of WM mask based on different FA thresholds of a control subject of the study. The mask obtained with a 0.2 FA threshold was found to most accurately discriminate white matter areas. FA: Fractional Anisotropy, IUGR: intrauterine growth restriction, WM: white matter, *p<0.05.
(TIF)

Figure S2 **Influence of the fractional anisotropy thresholds in the global analysis of DTI parameters on the mask of WM computed with different FA thresholds.** Control and IUGR average (A) Apparent Diffusion Coefficient, (B) Axial Diffusivity, (C) Radial Diffusivity, (D) Linearity coefficient, (E) Sphericity coefficient, (F) Planarity coefficient. Error bars depict ±1 standard deviation. *p<0.05.
(TIF)

Table S1 **Regional analysis of diffusion parameters in study groups.** IUGR: intrauterine growth restriction. Values are mean and standard deviation.
(DOC)

Table S2 **Correlations between neurobehavioral domains and fractional anisotropy in brain regions.** Cx: Cortex; GM: Gray matter; WM: White matter.
(DOC)

Video S1 **Illustrative video of neurobehavioral tests in cases and controls.**
(AVI)
