# Supplementary figures and images for: Neonatal Neurobehavior and Diffusion MRI Changes in Brain Reorganization Due to Intrauterine Growth Restriction in a Rabbit Model
Source: PLoS One. 2012 Feb 8;7(2):e31497. doi: 10.1371/journal.pone.0031497 (PMC3275591; doi:10.1371/journal.pone.0031497)

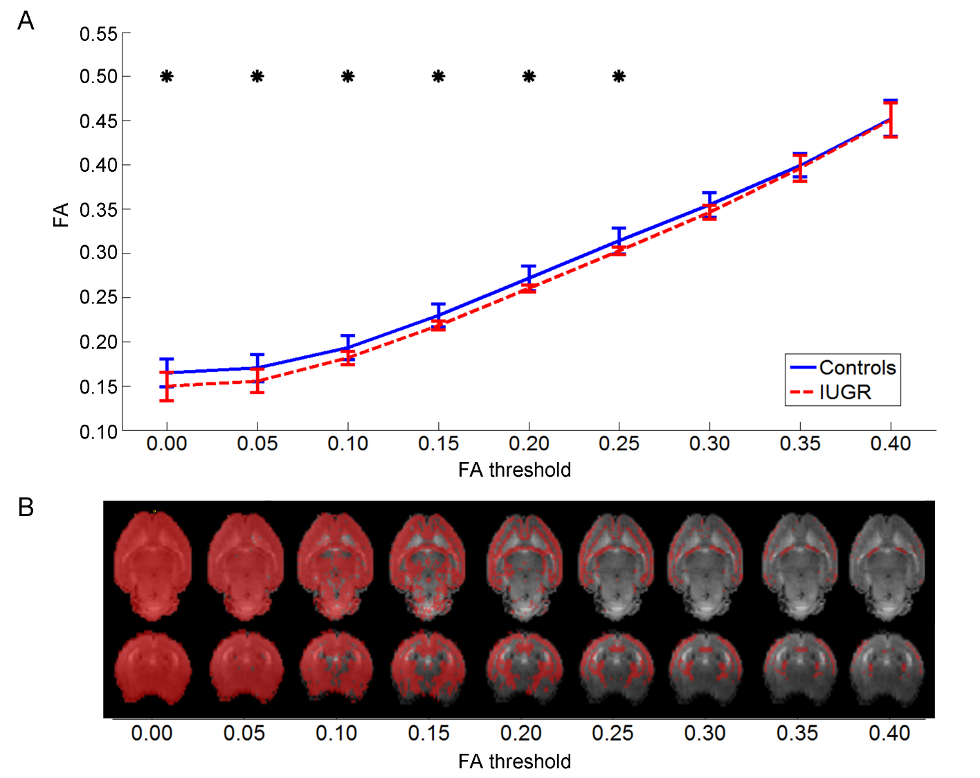

Supplement: Figure S1 — Fractional anisotropy thresholds in the global analysis. (A) Control and IUGR group distribution of average FA on the mask of WM computed with different FA thresholds. Error bars depict ±1 standard deviation. (B) Representative axial and coronal slices of WM mask based on different FA thresholds of a control subject of the study. The mask obtained with a 0.2 FA threshold was found to most accurately discriminate white matter areas. FA: Fractional Anisotropy, IUGR: intrauterine growth restriction, WM: white matter, *p<0.05. (TIF) [file pone.0031497.s001.tif]

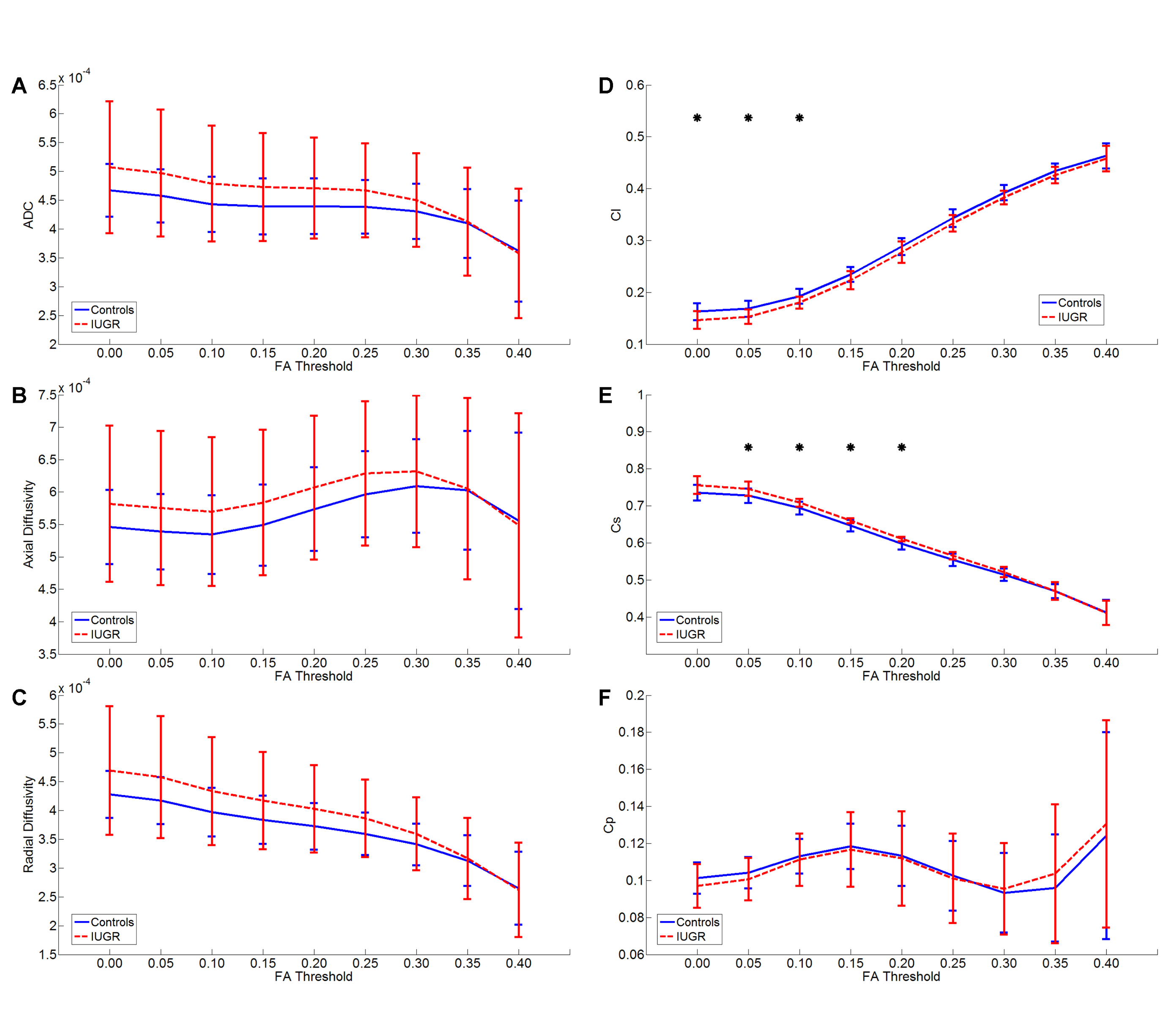

Supplement: Figure S2 — Influence of the fractional anisotropy thresholds in the global analysis of DTI parameters on the mask of WM computed with different FA thresholds. Control and IUGR average (A) Apparent Diffusion Coefficient, (B) Axial Diffusivity, (C) Radial Diffusivity, (D) Linearity coefficient, (E) Sphericity coefficient, (F) Planarity coefficient. Error bars depict ±1 standard deviation. *p<0.05. (TIF) [file pone.0031497.s002.tif]
