# Supplementary material for: Neonatal Neurobehavior and Diffusion MRI Changes in Brain Reorganization Due to Intrauterine Growth Restriction in a Rabbit Model
Source: PLoS One. 2012 Feb 8;7(2):e31497. doi: 10.1371/journal.pone.0031497 (PMC3275591; doi:10.1371/journal.pone.0031497)
Supplement: Table S1 — Regional analysis of diffusion parameters in study groups. IUGR: intrauterine growth restriction. Values are mean and standard deviation. (DOC) [file pone.0031497.s003.doc]

|  | **Fractional Anisotropy** | | | | **Apparent Diffusion Coefficient (x10^-3^mm^2^/s)** | | |
| --- | --- | --- | --- | --- | --- | --- | --- |
|  | Control n=10 | IUGR n=10 | | p | Control n=10 | IUGR n=10 | p |
| White matter structures | | | | | | | |
| Corpus callosum | 0.29 (0.04) | 0.28 (0.03) | | n.s. | 0.35 (0.07) | 0.34 (0.11) | n.s. |
| Left internal capsule | 0.26 (0.03) | 0.27 (0.04) | | n.s. | 0.37 (0.09) | 0.37 (0.08) | n.s. |
| Right internal capsule | 0.27 (0.02) | 0.25 (0.03) | | n.s. | 0.36 (0.10) | 0.38 (0.13) | n.s. |
| Left fimbria of hippocampus | 0.37 (0.05) | 0.35 (0.04) | | n.s. | 0.40 (0.09) | 0.40 (0.05) | n.s. |
| Right fimbria of hippocampus | 0.40 (0.04) | 0.36 (0.02) | | 0.048 | 0.40 (0.10) | 0.36 (0.07) | n.s. |
| Left corona radiata | 0.23 (0.04) | 0.23 (0.04) | | n.s. | 0.40 (0.10) | 0.39 (0.12) | n.s. |
| Right corona radiata | 0.23 (0.04) | 0.24 (0.04) | | n.s. | 0.41 (0.11) | 0.40 (0.08) | n.s. |
| Grey matter structures | | | | | | | |
| Cerebellar vermis | 0.13 (0.02) | 0.13 (0.02) | n.s. | | 0.47 (0.21) | 0.48 (0.15) | n.s. |
| Left cerebellar hemisphere | 0.11 (0.02) | 0.11 (0.03) | n.s. | | 0.57 (0.26) | 0.57(0.24) | n.s. |
| Right cerebellar hemisphere | 0.11 (0.02) | 0.12 (0.02) | n.s. | | 0.53 (0.19) | 0.54 (0.21) | n.s. |
| Left putamen | 0.18 (0.02) | 0.17 (0.02) | n.s. | | 0.39 (0.11) | 0.69 (0.10) | n.s. |
| Left caudate nucleus | 0.18 (0.02) | 0.17 (0.04) | n.s. | | 0.46 (0.10) | 0.46 (0.10) | n.s. |
| Left thalamus | 0.15 (0.02) | 0.14 (0.02) | n.s. | | 0.41 (0.11) | 0.41 (0.10) | n.s. |
| Right putamen | 0.20 (0.02) | 0.20 (0.03) | n.s. | | 0.38 (0.10) | 0.37 (0.10) | n.s. |
| Right caudate nucleus | 0.14 (0.02) | 0.13 (0.02) | n.s. | | 0.50 (0.18) | 0.51 (0.10) | n.s. |
| Right thalamus | 0.15 (0.03) | 0.14 (0.01) | n.s. | | 0.40 (0.09) | 0.41 (0.08) | n.s. |
| Left prefrontal cortex | 0.22 (0.03) | 0.20 (0.02) | n.s. | | 0.54 (0.17) | 0.52 (0.12) | n.s. |
| Right prefrontal cortex | 0.17 (0.03) | 0.17 (0.04) | n.s. | | 0.63 (0.15) | 0.62 (0.14) | n.s. |
