# Supplementary material for: Neonatal Neurobehavior and Diffusion MRI Changes in Brain Reorganization Due to Intrauterine Growth Restriction in a Rabbit Model
Source: PLoS One. 2012 Feb 8;7(2):e31497. doi: 10.1371/journal.pone.0031497 (PMC3275591; doi:10.1371/journal.pone.0031497)
Supplement: Table S2 — Correlations between neurobehavioral domains and fractional anisotropy in brain regions. Cx: Cortex; GM: Gray matter; WM: White matter. (DOC) [file pone.0031497.s004.doc]

|  |  | **Positive correlation** | **Negative correlations** |
| --- | --- | --- | --- |
| Posture | Cx: | Frontal, occipital, temporal |  |
|  | GM: | Thalamus |  |
|  | WM: | Anterior commissure, internal capsule, fimbria of hippocampus, olfactory tract |  |
| Righting reflex | Cx: | Occipital, temporal |  |
|  | GM: |  | Caudate nucleus |
|  | WM: | Anterior commissure |  |
| Tone | Cx: |  | Frontal, occipital, temporal |
|  | GM: | Caudate nucleus |  |
|  | WM: |  | Optic tract |
| Locomotion | Cx: | Frontal, occipital, temporal, insular |  |
|  | GM: | Hippocampus, thalamus |  |
|  | WM: | Fimbria of hippocampus, lateral lemniscus |  |
| Circular motion | Cx: | Frontal, occipital, temporal |  |
|  | GM: | Hippocampus, thalamus, caudate nucleus |  |
|  | WM: | Corona radiata, fimbria of hippocampus |  |
| Intensity | Cx: | Insular, temporal |  |
|  | GM: | Hippocampus, claustrum, |  |
|  | WM: | Olfactory tract, optic tract |  |
| Duration | GM: |  |  |
|  | WM: |  |  |
| Lineal movement | GM: | Hippocampus | Caudate nucleus, claustrum |
| Fore–hindpaw distance | Cx: |  | Frontal, occipital, temporal |
|  | GM: | Caudate nucleus | Hippocampus, thalamus |
|  | WM: |  | Fimbria of hippocampus, internal capsule, lateral lemniscus, optic tract |
| Sucking and swallowing | Cx: | Frontal, Occipital, Temporal |  |
|  | WM: | Fimbria of hippocampus |  |
| Head turn | Cx: | Frontal, Occipital, Temporal |  |
|  | GM: | Hippocampus, caudate nucleus, putamen, thalamus |  |
|  | WM: | Anterior commissure, corona radiata, internal capsule, fimbria of hippocampus |  |
| Smelling test | Cx: | Prefrontal, temporal |  |
|  | GM: |  |  |
|  | WM: | Olfactory tract |  |
| Smelling test time | Cx: |  | Prefrontal, temporal |
|  | GM: | Caudate nucleus |  |
